# PERCEIVED HOUSING PROBLEMS AND DEPRESSIVE SYMPTOMS AMONG MIDDLE-AGED AND OLDER AMERICANS

**DOI:** 10.1093/geroni/igad104.3517

**Published:** 2023-12-21

**Authors:** Peiyi Lu, Dexia Kong, Feifan Cao, Mack Shelley

**Affiliations:** Columbia University, New York City, New York, United States; The Chinese University of Hong Kong, Hong Kong, Hong Kong; Iowa State University, Ames, Iowa, United States; Iowa State University, Ames, Iowa, United States

## Abstract

**Background:**

Housing insecurity has been shown to be associated with worse mental health. However, previous studies mostly examined one dimension of housing insecurity (e.g., affordability) and few focused on older adults. This study examined the relationship of perceived housing problems with depressive symptoms among middle-aged and older Americans.

**Method:**

Longitudinal data from the Health and Retirement Study 2006-2018 were used. A total of 7,285 respondents (aged 50+ at baseline) were followed-up every 4 years. Respondents self-reported the status, severity, and duration of their housing problems. Depressive symptoms were assessed by the Center for Epidemiological Studies-Depression Scale (CESD). Mixed-effect models were used to examine the association between perceived housing problems and depressive symptoms, adjusting for individual-level, household-level, and housing-related confounders.

**Results:**

About 5%-7% of respondents had housing problems during every study visit and 5.67% of them experienced persistent housing problems over the study period of 12 years. Having housing problems was associated with higher risk of depressive symptoms (IRR=1.30, 95% CI=1.23, 1.36). A dose-response relationship was observed in the severity and duration of housing problems, with greater increase of depressive symptoms risk among those experiencing more severe or longer duration of housing problems. Results were similar when modelling the risk of elevated depressive symptoms using a binary measure (CESD score ≥ 4).

**Conclusion:**

Perceived housing problems were associated with worse mental health among middle-aged and older Americans. The dose-response pattern highlighted the importance of early intervention and consistent assistance to those experiencing housing problems.

